# Topological Analysis of the Language Networks of Ancient Traditional Chinese Medicine Books

**DOI:** 10.1155/2020/8810016

**Published:** 2020-12-10

**Authors:** Qunsheng Zou, Yinyan Wang, Zixin Shu, Kuo Yang, Jingjing Wang, Kezhi Lu, Qiang Zhu, Baoyan Liu, Runshun Zhang, Xuezhong Zhou

**Affiliations:** ^1^Beijing Key Lab of Traffic Data Analysis and Mining, School of Computer and Information Technology, Beijing Jiaotong University, Beijing 100044, China; ^2^Institute of Medical Intelligence, School of Computer and Information Technology, Beijing Jiaotong University, Beijing 100044, China; ^3^Data Center of Traditional Chinese Medicine, China Academy of Chinese Medical Sciences, Beijing 100700, China; ^4^Guang'anmen Hospital, China Academy of Chinese Medicine, Beijing 100700, China

## Abstract

This study aims to explore the topological regularities of the character network of ancient traditional Chinese medicine (TCM) book. We applied the 2-gram model to construct language networks from ancient TCM books. Each text of the book was separated into sentences and a TCM book was generated as a directed network, in which nodes represent Chinese characters and links represent the sequential associations between Chinese characters in the sentences (the occurrence of identical sequential associations is considered as the weight of this link). We first calculated node degrees, average path lengths, and clustering coefficients of the book networks and explored the basic topological correlations between them. Then, we compared the similarity of network nodes to assess the specificity of TCM concepts in the network. In order to explore the relationship between TCM concepts, we screened TCM concepts and clustered them. Finally, we selected the binary groups whose weights are greater than 10 in *Inner Canon of Huangdi* (ICH, 黄帝内经) and *Treatise on Cold Pathogenic Disease* (TCPD, 伤寒论), hoping to find the core differences of these two ancient TCM books through them. We found that the degree distributions of ancient TCM book networks are consistent with power law distribution. Moreover, the average path lengths of book networks are much smaller than random networks of the same scale; clustering coefficients are higher, which means that ancient book networks have small-world patterns. In addition, the similar TCM concepts are displayed and linked closely, according to the results of cosine similarity comparison and clustering. Furthermore, the core words of *Inner Canon of Huangdi* and *Treatise on Cold Pathogenic Diseases* have essential differences, which might indicate the significant differences of language and conceptual patterns between theoretical and clinical books. This study adopts language network approach to investigate the basic conceptual characteristics of ancient TCM book networks, which proposes a useful method to identify the underlying conceptual meanings of particular concepts conceived in TCM theories and clinical operations.

## 1. Introduction

As a traditional medicine with medical theories and concepts mainly established thousands of years ago, TCM has abundance of high-value ancient books written or printed in the form of Chinese classical binding before 1912, which conceive important TCM theories and concepts and the clinical principle for disease diagnosis and treatment [1, 2]. Although many TCM antecessors performed significant theoretical investigations to digest the knowledge employed in these books, which has promoted the advances of contemporary clinical practical solutions for the managing of various complicated diseases in real-world clinical settings [3], it is particularly important to investigate the language characteristics of ancient TCM books, which will help understand the theoretical knowledge exactly expressed in those texts [4]. However, little research was conducted to understand the language regularities of the key TCM concepts (e.g., Yin, Yang, and Qi) in these ancient books using computational linguistics and complex network approaches [5].

Complex network has been developed as a mainstream approach for investigating the regularities in the fields with complex phenomena, such as social science, biological science, and linguistics [6–8]. For this approach, network or graph consisting of nodes and links is the form to represent the structures of the related systems. Due to the complex organization and interactions between various medical entities, complex network approaches have been used for exploring the rules of associations between herbs, symptoms, syndromes, and human meridians [9–12]. However, rare work was conducted on the analysis of language regularities of TCM books by complex network approaches [13].

In this paper, firstly, we constructed directed networks from the full texts of ancient TCM books. Then, we analyzed the statistical characteristics of the networks, identified the centrality patterns of core TCM concepts, and explored the similarities and differences between different ancient books. In addition, we demonstrated what the diversity of the concepts, such as “Qi (气)”, “Yin (阴)”, “Yang (阳)”, “Xie (泻)”, and “Li (痢)”, in Chinese medicine, and what special conceptual meanings they would have.

## 2. Materials and Methods

### 2.1. Dataset of 80 Ancient TCM Books

The data we used was derived from texts of 80 ancient Chinese medicine books (Table 1 shows a typical collection of 30 books), with an emphasis on the analysis of the books of *Inner Canon of Huangdi* (ICH) and *Treatise on Cold Pathogenic Disease* (TCPD). For example, ICH contains 189,984 characters and TCPD contains 43,331 characters. Data cleaning was performed to remove the characters in ancient texts except for Chinese and periods and separated the whole text into sentences. For example, ICH and TCPD, after cleaning the data of these two ancient books, we obtained 6,237 sentences and 1,366 sentences, respectively.

### 2.2. Language Network Construction Using 2-Gram Model

**Figure 1:**
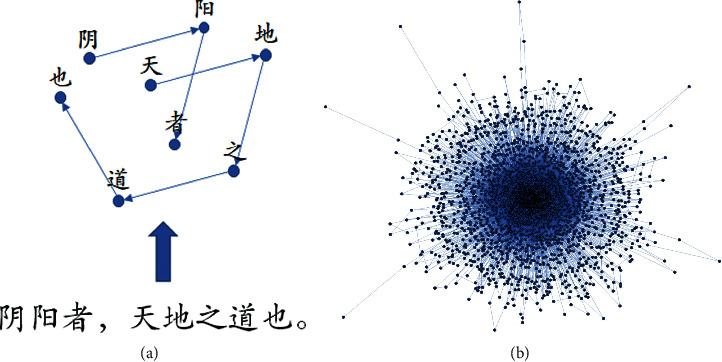
(a) An example of ancient TCM book network. (b) The language network of ICH.

In the field of computational linguistics, *n*-gram is a widely used method to model natural language; particularly, an *n*-gram is a contiguous sequence of *n* items from a given sequence of text [14, 15]. Here, we used 2-gram model to obtain the sequential links of characters in ancient TCM books. Given a sentence, we would generate a directed path with characters as nodes and the sequential associations between them as links. When all the sentences of a given book were processed, we would obtain a weighted directed language network, in which the number of identical sequential associations is considered as the weight of the link. For example, the sentence “阴阳者, 天地之道也” in ICH can be processed to a directed path (Figure 1(a)) [16]. We have built the language networks for all the 80 ancient TCM books. In particular, the network of ICH contained 2,367 nodes and 35,502 directed links (see Figure 1(b)).

### 2.3. Basic Network Characteristics

The number of links connected to each node in TCM book networks, that is, the degree of the node [17]. We counted the degree of each node in the network and figured up the number of nodes with the same degree and attempted to find out whether the degree distributions of ancient book networks are consistent with the power law [18]. By calculating the average path length [19] and clustering coefficients [20] of networks, we judged whether these networks possess the small-world property. Thus,

(1) $$\begin{array}{c} l_{G}=\frac{1}{n\left(n-1\right)}\cdot \sum_{i=j}d\left(v_{i},v_{j}\right), \end{array}$$

where *l*_*G*_ is the average path length of graph *G*, *n* is the number of nodes, and *d*(*v*_*i*_, *v*_*j*_) denotes the shortest distance between *v*_*i*_ and *v*_*j*_. When *v*_*j*_ cannot be reached from *v*_*i*_, *d*(*v*_*i*_, *v*_*j*_) = 0. Moreover, clustering coefficients are acquired by

(2) $$\begin{array}{c} C_{i}=\frac{\left|\left(e_{jk}:v_{j},v_{k}\in N_{i},e_{jk}\in E\right)\right|}{d_{i}\left(d_{i}-1\right)},\\ \bar{C}=\frac{1}{n}\overset{n}{\underset{i=1}{\sum }}C_{i}. \end{array}$$

Consider *C*_*i*_ as the local clustering coefficient of node *i*, where *d*_*i*_ is the degree of node *i*, *N*_*i*_ is a set of nodes which immediately connected with node *i*, *E* is defined as a set of edges in graph *G*, and *e*_*jk*_ is the edge of nodes *j* and *k*. Then, all *C*_*i*_ were summed and averaged to get the average clustering coefficient $\bar{C}$.

### 2.4. Homogeneity of the Centrality of Similar TCM Concepts in the Language Network

In TCM theories, there are hundreds of similar basic concepts (often in the form of a single character word), such as “Yin and Yang (阴阳)”, “the five elements (五行)”, and “the five internal organs (五脏)”, which are essential for TCM theories and clinical solutions [21]. We supposed that this kind of essentiality or importance of the concepts could be captured by vector representation of nodes in the directed language networks. In addition, we assumed that for those similar concepts, they would finally have similar centralities; that is, the similar concepts would display the same degree of centrality homogeneity compared with the random sets of concepts. The vector representation of each node in book network is calculated by the Node2Vec framework which learns low-dimensional representations for nodes in a graph [22]. To investigate the homogeneity phenomenon of these similar concepts, we proposed the following methods to differentiate between similar concepts and their random controls:

(3) $$\begin{array}{c} \text{sim}=\cos\left(\theta \right)=\frac{A\cdot B}{\left\|A\right\|\left\|B\right\|}=\frac{\sum_{i=1}^{n}a_{i}\times b_{i}}{\sqrt{\sum_{i=1}^{n}{\left(a_{i}\right)}^{2}}\times \sqrt{\sum_{i=1}^{n}{\left(b_{i}\right)}^{2}}}, \end{array}$$

where *A* and *B* are the low-dimensional representation of nodes, *a*_*i*_ and *b*_*i*_ are their components, and *i*=1,  2,  …,  *n*, *n* is the dimension of vectors.

Determine *S* as a set of similar concepts and *R* as the set of another nodes in network. The similarity of concepts in groups will be defined by sim_*i*, *j*_ and similarity between in-group concepts and out-of-group concepts will be defined by sim_*i*, *k*_, where *i*, *j* ∈ *S* and *k* ∈ *S*. If our hypothesis is correct, then sim_*i*, *j*_ is generally greater than sim_*i*, *k*_.

### 2.5. *t*-Test on Similarity Sequences

Through the above method, we gained the difference between similarity of each basic concept and random concept. However, the current results were only relative to a single basic concept, which did not indicate that similar concepts are homogeneous to some extent. There is a way to solve this problem, named the Student *t*-test, which is often used to assess whether the means of two classes are statistically different from each other by calculating a ratio between the difference of two class means and the variability of the two classes [23, 24]. In this way, we can verify the homogeneity of basic concepts indirectly through the results of *t*-test. Whereupon, we combined results of each basic concept into similarity sequence *M* and results of random concepts into similarity sequence *N*. After performing *t*-test on these two sequences, if *P* value is less than 0.05, we believe these basic concepts are similar to each other and consistent with homogeneity.

### 2.6. Identifying the Concept Clusters

It is well recognized that the complex networks like language network often hold a kind of community structures with some subnetworks involving dense links while sparse links outside those subnetworks. These subnetworks, which are considered as network clusters or communities, would deliver domain meaningful knowledge for further investigation. To detect the concept clusters or communities in the TCM language network, we applied the Fast Unfolding Algorithm (FUA) which was a well-known community detection method based on modularity [25] to detect the communities of a given network by

(4) $$\begin{array}{c} Q=\frac{1}{2m}\sum_{ij}\left[W_{ij}-\frac{k_{i}k_{j}}{2m}\right]\delta \left(C_{i},C_{j}\right). \end{array}$$

Think of *Q* as the modularity of the entire network, where *m*=(1/2)∑_*ij*_*W*_*ij*_ represents the sum of the weights of all the edges in the network, *W*_*ij*_ is the weight between node *i* and node *j*, *k*_*i*_=∑_*j*_*W*_*ij*_ is the sum of the weights of the edges which connected to node *i*, and *C*_*i*_ indicates the community which node *i* is assigned to. The value of *δ*(*C*_*i*_, *C*_*j*_) is 0 or 1; when *δ*(*C*_*i*_, *C*_*j*_)=1, it means node *i* and node *j* are in the same community; otherwise, node *i* and node *j* are not in the same community. Then, iteratively making the modularity reach the maximum value, the final clustering results were obtained.

## 3. Results

### 3.1. Basic Characteristics of TCM Language Network

**Figure 2:**
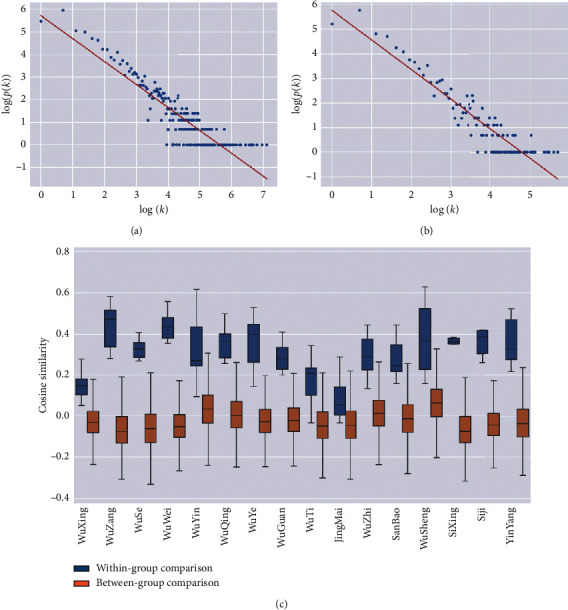
The degree patterns of TCM language networks. (a) Degree distribution of ICH. (b) Degree distribution of TCPD. (c) Cosine similarity of with-group comparison and between-group comparison.

It is observed that the degree distribution of ancient Chinese medicine nodes is consistent with the power law distribution [26, 27] (Figures 2(a) and 2(b)), which means that although most characters were rarely used together with other characters; there are some “Hub” characters, such as “Qi”, “Yin”, and “Yang”, connecting to a various number of characters in the sentences. We listed the basic network features of the 30 typical TCM books which are divided into 10 categories. It can be found that the power exponents of books are close to 1.0; the biggest one is 1.3246 of the *Valuable Prescriptions for Emergency*, and the smallest one is 0.9499 of the *Medical Complete Book, Ancient and Modern*. The node degree distributions of these ancient books follow

(5) $$\begin{array}{c} p\left(k\right)\propto k^{-\gamma }, \end{array}$$

where *k* is the degree of the node, *p*(*k*) is the ratio of the number of nodes with a degree of *k* to the total number of nodes, and *γ* is the power exponent which floats above and below 1.

In addition, the average path lengths of these networks are around 3, in which the largest one is 3.819 and the smallest one is 2.727. The clustering coefficients are distributed between 0.1 and 0.3 (Table 2). Comparing these ancient books with random networks of the same scale, it is found that their average path lengths are smaller than random networks and the clustering coefficients are larger than random networks. It means that TCM language networks conform to the small-world pattern [7].

### 3.2. Topological Homogeneity of TCM Basic Concept Groups

**Figure 3:**
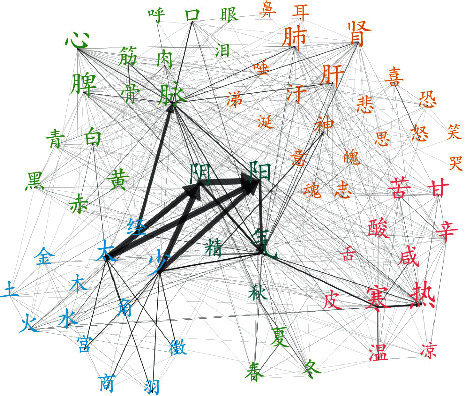
TCM concepts clusters of *Inner Canon of Huangdi.*

To validate the power of complex network approach to differentiate the semantic groups of basic TCM concepts from the language network, we calculated the cosine similarity of each node vector of 16 basic TCM concept groups (Table 3). We assumed that the basic TCM concept groups, such as these concepts of five elements, would have similar values for the centrality measures, which would reflect their similar semantic importance in the language network from the topological measures. The results showed that most of the basic TCM concept groups in basic theoretical books (e.g., ICH) are more similar to each other than those of random controls (Table 4), which indicated that these basic TCM concept groups display a kind of linking homogeneity reflecting their close category semantic similarities. For example, the five elements concept category includes Mu, Huo, Tu, Jin, and Shui as closely related members. We found that, in the ICH book (Figure 2(c)), the cosine similarity of these five elements ranges from 0.0499 to 0.2786 with a rather high value of very narrow variance (mean: 0.1498 + std: 0.0669). This demonstrated the central role of the concepts in the five elements category for TCM and the categorical homogeneity of these five concepts. In addition, these category similar concepts could be identified by community detection methods due to their similar connection patterns in the context of network. For example, in ICH network, using FUA (see methods), we could identify the concept groups as same communities, such as “Yin and Yang”, “the five elements”, “the five notes”, and “the five colors” from the whole network (Figure 3).

However, the results were different for those clinical books (e.g., TCPD). The cosine similarity of the basic TCM concept groups did not tend to show homogeneous patterns. This might be due to the differently focused subjects of these books. For example, TCPD is mainly focusing on the manifestations of six types of syndromes and their regularities of herb treatment.

### 3.3. Diversity of TCM Language Networks

**Figure 4:**
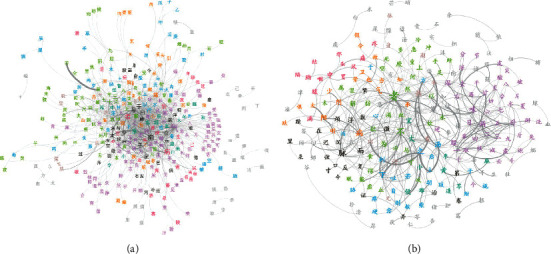
Two cases of TCM language networks. (a) The key TCM concepts of *Inner Canon of Huangdi*. (b) The key TCM concepts of *Treatise on Cold Pathogenic Diseases.*

To further investigate the distinct topological patterns involved in different TCM language networks, we screened the links whose weights are >10 in ICH and TCPD and regarded the related nodes (Chinese characters) as key concepts in these two books (Figure 4). It is illustrated that the key concepts in ICH mainly include the basic theoretical characters in TCM, such as “Yin/Yang”, “the five elements” and its associated concepts, quantifiers, emotions, and pulse (Figure 4(b)). In contrast, although several basic theoretical concepts, such as “Yin/Yang”, are still included in TCPD as the key concepts, most of the others are related to herb prescriptions and symptoms (Figure 4(b)). These results indicated the distinct category of knowledge delivered in these two books. It is well known that ICH ensembles the basic theories of TCM, while TCPD is recognized as a representative clinical book focusing on disease manifestations, pathologies, and their corresponding herb prescriptions.

### 3.4. Exploring the Specific Semantic Intensions of Core TCM Concepts

**Figure 5:**
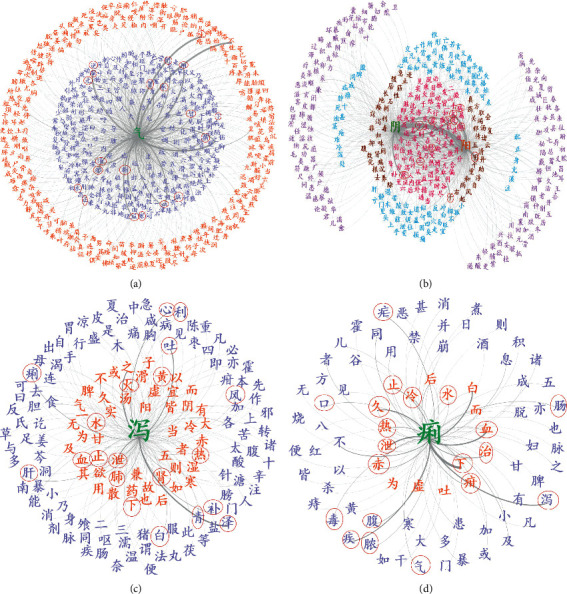
(a) The network centered on “Qi.” (b) The network centered on “Yin” and “Yang.” (c) The network centered on “Xie.” (d) The network centered on “Li.”

To identify the specific meaning of a given concept, we would like to see what exactly words or phrases it occurred. The TCM language network could give help to this investigation. It is well known that some basic concepts, such as “Qi” and “Yin and Yang”, are of great significance to TCM; however, the connotations of these concepts are rather complicated [28–30]. We constructed an integrated language network with various character triples derived from 30 ancient TCM books, which finally consists of 6118 nodes and 381467 links. Here, we extracted the 1-order neighborhood of a given node and took four concepts, namely, “Qi”, “Yin/Yang”, “Xie”, and “Li” for demonstrations (Figure 5). It is interesting that for the basic concept of “Qi”, there are about 1864 characters (nodes) directly connecting to this node, in which the characters, such as “Yang”, “Xue”, “Yin”, “Yuan”, “Zheng”, and “Jing” together with “Qi” represent the main types of “Qi” recognized in TCM theories. The other connecting characters to “Qi” obtained the various manifestations and pathologies, such as “XiaQi”, “QiNi” and “QiXu”; “XieQi”, “HanQi”. Although these concepts are usually adopted by professional TCM practitioners, our network results could grasp and demonstrate the global connecting characters for TCM researchers. Similarly, we obtained 1736 characters related to “Yin and Yang”, which could form different “Yin and Yang”-related basic concepts, such as meridian-related concepts (e.g., “TaiYin”, “TaiYang”, “YangMai”, and “YinMai”), syndrome-related concepts (e.g., “YinXu” and “YangXu”). For the character “Xie”, our network approach clearly showed two types of semantics involved. One type of concept is related to different manifestations, such as “XieXie”, “TuXie”, “ShuiXie”, and “FengXie”. Another type of concept is related to the principles for prescriptions including “XieXin” and “XieHuo”. However, the concepts related to “Li” are only associated with disorders or diseases, such as “XueLi”, “NueLi”, “GanLi”, “LiChang”, and “LenLi” ^1^. The rigorous evaluation of these related concepts would help with the precise understanding of the manifestations related to “Li” and improve distilling the high-value disease or prescription knowledge from TCM ancient literatures.

## 4. Discussion

Medical concepts constitute the basic knowledge framework of TCM theories devoted to the clinical observation of the complicated manifestations and their understanding of the underlying pathologies from TCM perspectives. Therefore, the development of TCM terminologies even with international translations is an important task in TCM field [31–33]. However, as most TCM concepts derived from ancient textbooks, it is difficult for contemporary practitioners to definitely grasp the whole meanings and connotations in the framework of TCM theories, in which the semantic diversity of a specific TCM concept is one of the key issues. Language network proposes an efficient approach to investigate the semantic properties of concepts of words in large-scale text corpora [34]. The application of complex network in linguistics has made it possible for us to adopt real network analysis tools in ancient TCM book studies. Unfortunately, the current researchers of TCM are mostly those with medical science background, who usually concern themselves with clinical medicine. It lacks some approaches, which focus on ancient TCM books' concepts, are not only helpful to the research on basic theory of TCM but also helpful to nonprofessionals understanding the basic concepts.

Ancient TCM books are carriers of Chinese medicine knowledge and have great significance for the entire Chinese civilization [35]. In this paper, we analyzed TCM books in the form of network and explored some characteristics of ancient language networks. First of all, the node degree distributions, average path lengths, and clustering coefficients of the networks showed that TCM character-language networks follow a kind of scale-free and small-world networks. Secondly, we analyzed the basic concepts in ancient TCM book networks and found that these concepts play special roles in language networks. Furthermore, we extracted key TCM concepts of each book and found that the key concepts in different categories of ancient books have obvious differences. Finally, we drew a conclusion that Chinese medicine concepts such as “Qi” have rich medical connotations in ancient books.

There are several limitations in our manuscript. Firstly, we only constructed dozens of language networks, which might influence the extensions of the obtained results to more general context. In addition, the character-based 2-gram modeling also limits the investigation capability of the language network for semantic issues. Secondly, although most TCM basic concepts could be grasped by single character (e.g., Qi), there exist many key concepts, such as those of acupuncture points, herbs, and disorders, which would necessarily be represented by words or phrases to further explore their semantic regularities. Furthermore, it is notable that network approach is adept in investigating the global patterns of a given domain, which could be combined with other data analysis methods (e.g., association rules) to generate more specific results to deliver TCM meaningful knowledge.

## 5. Conclusion

In summary, we found that the degree distribution of ancient TCM book networks is consistent with power law distribution and small-world patterns. In addition, similar concepts in ancient books are displayed and linked closely. Moreover, we realized that there are essential differences in language and conceptual patterns between theoretical and clinical books. To sum up, the exploration of ancient TCM books provides an effective method to identify the underlying conceptual meanings of particular concepts conceived in TCM theories and clinical operations.

## Figures and Tables

**Table 1:** The power exponents of TCM language networks.

Category	Book	Power exponent
Medical classics	*ICH*	1.0169
	*CMP*	1.1703
	*YJYZ*	0.9864
Basic theory	*HSZZJ*	1.2469
	*YXQY*	1.1514
	*WXDY*	1.1367
Typhoid	*TCPD*	1.2000
	*SGC*	1.2169
	*ZQZSHL*	1.0271
Diagnostic methods	*CBZN*	1.2360
	*YDXY*	1.1020
	*ZJSY*	1.1778
Acupuncture and massage	*A-B-CAM*	1.0533
	*ZJZN*	1.3123
	*ZJZSJ*	1.1251
Materia medica	*EMM*	1.1205
	*CNCMM*	1.1880
	*SNCMM*	1.2647
Medical formulary	*VPE*	**1.3246**
	*BZYBY*	1.1889
	*JYF*	1.2502
Clinical examination	*BQHB*	1.2099
	*SBOC*	1.0794
	*CDP*	1.0983
Health preserving	*BPZNP*	1.1610
	*BPZWP*	1.1988
	*YSML*	1.3086
Comprehensive work	*GJMYHC*	1.0098
	*MCB-A-M*	**0.9499**
	*EM*	0.9586

*Note*. *ICH: Inner Canon of Huangdi*; *CMP: Classic on 81 Medical Problems* (黄帝八十一难经); *YJYZ: Yijing Yuanzhi* (医经原旨); *HSZZJ: Huashi Zhongzang Jing* (华氏中藏经); *YXQY: Yixue Qiyuan* (医学启源); *WXDY: Wuxing Dayi* (五行大义); *TCPD: Treatise on Cold Pathogenic Diseases*; *SGC: Synopsis of Golden Chamber* (金匮要略); *ZQZSHL: Zhangqingzi Shanghan Lun* (张卿子伤寒论); *CBZN: Chabing Zhinan* (察病指南); *YDXY: Yideng Xuyan* (医灯续焰); *ZJSY: Zhenjia Shuyao* (诊家枢要); *A-B CAM: A-B Classic of Acupuncture and Moxibustion* (针灸甲乙经); *ZJZN: Zhenjing Zhinan* (针经指南); *ZJZSJ: Zhenjiu Zisheng Jing* (针灸资生经); *EMM: Essentials of Matea Medica* (本草备要); *CNCMM: Collective Notes to the Canon of Materia Medica* (本草经集注); *SNCMM: Shennong's Classic of Materia Medica* (神农本草经); *VPE: Valuable Prescriptions for Emergency* (备用千金要方); *BZYBY: Buzhi Yi Biyao* (不知医必要); *JYF: Jiyan Fang* (集验方); *BQHB: Bian Que Heart Book* (扁鹊心书); *SBOC: Secret Book of Orchid Chamber* (兰室秘藏); *CDP: Confucians' Duties to Parents* (儒门事亲); *BPZNP: Baopuzi Neipian* (抱朴子内篇); *BPZWP: Baopuzi Waipian* (抱朴子外篇); *YSML: Yangsheng Milu* (养生秘录); *GJMYHC: Gujin Mingyi Huicui* (古今名医汇粹); *MCB A-M: Medical Complete Book, Ancient and Modern* (古今医统大全); and *EM: Elementary Medicine* (医学入门).

**Table 2:** Basic topological characteristics of TCM ancient books.

Book	Average path length (APL)	APL random	*P* value	Average clustering coefficients (ACC)	ACC random	*P* value
*ICH*	3.036	2.973	<2.23*E* − 308	0.249	0.0077	<2.23*E* − 308
*CMP*	3.590	4.012	<2.23*E* − 308	0.123	0.0092	7.7*E* − 71
*YJYZ*	2.912	2.957	<2.23*E* − 308	0.297	0.0077	<2.23*E* − 308
*HSZZJ*	3.674	3.219	<2.23*E* − 308	0.097	0.0078	9.44*E* − 129
*YXQY*	3.434	3.292	<2.23*E* − 308	0.140	0.0076	3.7*E* − 164
*WXDY*	3.329	2.967	<2.23*E* − 308	0.154	0.0076	1.9*E* − 298
*TCPD*	3.584	3.367	<2.23*E* − 308	0.119	0.0077	5.06*E* − 120
*SGC*	3.548	3.340	<2.23*E* − 308	0.107	0.0067	3.34*E* − 127
*ZQZSHL*	3.199	3.164	2.77*E* − 167	0.202	0.0074	6.57*E* − 276
*CBZN*	3.702	3.509	<2.23*E* − 308	0.098	0.0073	4.76*E* − 91
*YDXY*	3.103	2.847	<2.23*E* − 308	0.188	0.0075	<2.23*E* − 308
*ZJSY*	3.505	3.712	<2.23*E* − 308	0.135	0.0082	2.03*E* − 89
*A-B CAM*	3.096	3.060	<2.23*E* − 308	0.232	0.0074	<2.23*E* − 308
*ZJZN*	3.749	3.472	<2.23*E* − 308	0.085	0.0069	7.3*E* − 74
*ZJZSJ*	3.275	3.062	<2.23*E* − 308	0.158	0.0077	5.62*E* − 275
*EMM*	3.227	2.884	<2.23*E* − 308	0.154	0.0074	<2.23*E* − 308
*CNCMM*	3.327	2.929	<2.23*E* − 308	0.144	0.0074	<2.23*E* − 308
*SNCMM*	3.622	2.935	<2.23*E* − 308	0.112	0.0076	4.21*E* − 209
*VPE*	**3.819**	3.259	<2.23*E* − 308	**0.078**	0.0075	3.66*E* − 99
*BZYBY*	3.406	3.064	<2.23*E* − 308	0.127	0.0075	5.41*E* − 231
*JYF*	3.521	3.086	<2.23*E* − 308	0.106	0.0075	3.49*E* − 180
*BQHB*	3.488	3.009	<2.23*E* − 308	0.116	0.0076	2.49*E* − 197
*SBOC*	3.302	3.259	<2.23*E* − 308	0.158	0.0075	3.06*E* − 206
*CDP*	3.143	2.875	<2.23*E* − 308	0.191	0.0076	<2.23*E* − 308
*BPZNP*	3.185	2.831	<2.23*E* − 308	0.168	0.0073	<2.23*E* − 308
*BPZWP*	3.167	2.877	<2.23*E* − 308	0.159	0.0074	<2.23*E* − 308
*YSML*	3.814	3.461	<2.23*E* − 308	0.086	0.0077	2.05*E* − 75
*GJMYHC*	3.043	2.932	<2.23*E* − 308	0.244	0.0072	<2.23*E* − 308
*MCB A-M*	**2.727**	2.767	<2.23*E* − 308	**0.319**	0.0075	<2.23*E* − 308
*EM*	2.843	2.795	<2.23*E* − 308	0.270	0.0075	<2.23*E* − 308

*t*-test was used to compare the real and random measures.

**Table 3:** The mean and standard deviation of cosine similarity of each node of 16 basic TCM concept groups in ICH.

Concept groups	Mean	Standard deviation
WuXing (五行)	0.1498	0.0669
WuZang (五脏)	0.4378	0.0996
WuSe (五色)	0.3407	0.0712
WuWei (五味)	0.4363	0.0633
WuYin (五音)	0.3351	0.1487
WuQing (五情)	0.3581	0.0756
WuYe (五液)	0.3587	0.1144
WuGuan (五官)	0.2855	0.0681
WuTi (五体)	0.1692	0.1104
WuZhi (五志)	0.3144	0.1496
WuSheng (五声)	0.3703	0.1674
SiXing (四性)	0.3496	0.0767
SiJi (四季)	0.3963	0.1243
YinYang (阴阳)	0.3606	0.1178
SanBao (三宝)	0.2824	0.0969
JingMai (经脉)	0.0904	0.1222

**Table 4:** The difference between basic TCM concept groups and random controls in ancient books (by *t*-test).

Book	*P* value
*ICH*	1.08*E* − 12^c^
*CMP*	0.064492
*YJYZ*	6.28*E* − 23^c^
*HSZZJ*	0.418429
*YXQY*	0.361999
*WXDY*	3.58*E* − 23^c^
*TCPD*	0.856763
*SGC*	0.807354
*ZQZSHL*	0.182582
*CBZN*	0.650913
*YDXY*	0.239566
*ZJSY*	0.677128
*A-B-CAM*	3.93*E* − 06^c^
*ZJZN*	0.061295
*ZJZSJ*	0.018853^a^
*EMM*	0.086299
*CNCMM*	0.575891
*SNCMM*	0.059049
*VPE*	5.95*E* − 06^c^
*BZYBY*	0.012146^a^
*JYF*	0.027935^a^
*BQHB*	0.000277^c^
*SBOC*	0.499433
*CDP*	0.281959
*BPZNP*	4.97*E* − 30^c^
*BPZWP*	1.10*E* − 27^c^
*YSML*	0.740321
*GJMYHC*	3.84*E* − 06^c^
*MCB-A-M*	0.008839^b^
*EM*	0.572089

*Note*. *P* value < 0.05 means that most of the basic TCM concept groups in this book are more similar to each other than random controls. ^a^*P* value < 0.05, ^b^*P* value < 0.01, and ^c^*P* value < 0.001.

## Data Availability

Data used in this paper are found at https://gitee.com/zouqunsheng/ancient-tcm-books.git.
